# The Effect of Pet Remedy on the Behaviour of the Domestic Dog (*Canis familiaris*)

**DOI:** 10.3390/ani6110064

**Published:** 2016-10-25

**Authors:** Sienna Taylor, Joah Madden

**Affiliations:** 1 Animal Behaviour and Welfare Research Group, Department of Animal and Land Sciences, Hartpury University Centre, Hartpury, Gloucestershire GL19 3BE, UK; 2 School of Psychology, University of Exeter, Washington Singer Building, Perry Road, Exeter EX4 4QG, UK; j.r.madden@exeter.ac.uk

**Keywords:** stress, dogs, sighing, Pet Remedy, multivariate regression, welfare

## Abstract

**Simple Summary:**

In this placebo controlled study, we exposed 28 dogs to Pet Remedy (a natural stress relief product) to investigate whether Pet Remedy lowered stress-affected behaviour. No statistically significant differences were found when dogs were exposed to Pet Remedy or the placebo condition. We suggest that Pet Remedy, in this particular study, did not have a discernible effect on changes in behaviour. Further research determining the effects of Pet Remedy would be beneficial.

**Abstract:**

Stress-affected behaviour in companion animals can have an adverse effect on animal health and welfare and their relationships with humans. This stress can be addressed using chemical treatments, often in conjunction with behavioural therapies. Here, we investigated the efficacy of one commercial pharmacological intervention, Pet Remedy, advertised as a natural stress relief product for mammals. We aimed to see whether the product lowered stress-affected behaviour in dogs placed in a non-familiar environment. Behavioural responses of 28 dogs were video recorded using a double-blind, placebo-controlled, and counterbalanced repeated measures design. Dogs were exposed to both a placebo and Pet Remedy plug-in diffuser for 30 min with an intervening period of approximately 7 days between conditions. Multivariate regression analysis identified no significant differences in behaviour in either the Pet Remedy or placebo condition. In conclusion, in the current study, Pet Remedy did not reduce behavioural indicators indicative of a stress response. To determine the effects of Pet Remedy, future research using a larger sample size and controlling for breed would be beneficial.

## 1. Introduction

The vast majority of domestic dogs (*Canis familiaris*) will, at some point in their lives, experience stress. Stressors include social isolation from humans and other dogs [1], confinement [2], loud or unfamiliar noises [3], transportation [4] and novel environments that are both uncontrollable and unpredictable (e.g., kennels) [1]. Chronic and persistent stress can lead to destructive behaviour; excessive vocalisation, inappropriate elimination and self-mutilation, particularly in response to social detachment from prolonged bonds between both dogs and humans [5,6]. Many of these behaviours are deemed by dog owners as undesirable with behavioural problems reported to occur in up to 90% of dogs [7]. As a result, these dogs are frequently relinquished to shelters [8] or euthanised [9].

Stress can be broadly defined as, “a real or interpreted threat to the physiological or psychological integrity of an individual that results in physiological and/or behavioural responses...” [10]. Typically, novel, uncontrollable situations elicit changes in behavioural, cardiovascular and endocrine parameters indicative of a stress response [11]. Regardless of whether threats are real or interpreted, individuals respond to stress differently, suggesting that individual dogs may have different “coping styles” in how they respond to and deal with potentially stressful situations [12]. Contrasts in studies whereby responses vary substantially between individuals have been reported. For example, many dogs become hyper-active and perform increased levels of locomotory or oral behaviours in response to acute stress [13]. Alternatively, such behaviour may become subdued [14]. These variations are due to individual differences in behaviour such as temperament [15] or coping styles [12], which can arise through a combination of interactions between genetic factors (e.g., breed and sex [16]) and environmental factors (e.g., experience [17], rearing environment [18] and neuter status [16]). Recording behaviours indicative of stress has been used successfully as a measure of welfare in dogs [14] and is an inexpensive and non-invasive method.

In this study, a comprehensive range of behaviours were recorded, including previously reported behaviours indicative of stress as a result of physical and social isolation such as high levels of sighing, circling and recurrent yawning (a sign of discomfort, uneasiness or tension) [19]. Many of these indicators are performed alongside panting, trembling and lip licking and in combination are deemed as a response to acute stressors such as confinement [11,20,21]. Behaviours that were indicative of resting and relaxation were also included such as lying down and sitting [22]. 

Common attempts to manage stress-related behavioural problems in dogs include sedation [23] which can impair welfare to some extent, mainly by inhibiting freedom to express normal behaviour. Alternatively, pharmacological interventions in conjunction with behavioural desensitisation training may be used and have been successfully used in combination in previous studies to reduce the occurrence of behavioural problems [24,25]. These may be based on synthetic compounds. For example, one of these, clomipramine an anti-depressive drug, has successfully reduced stress in humans [26] as well as companion animals, however the drug also induced secondary negative symptoms such as vomiting in dogs [27]. A second product, DAP (Dog Appeasing Pheromone) has had some success in reducing canine stress but did not reduce aggression [28] or amplitude of barking [29] and may increase undesirable behaviours [30]. Pharmacological interventions may also be based on natural products and their use has grown in popularity amongst pet owners and veterinarians [31]. Lavender (*Lavandula angustifolia*) and chamomile (*Anthennis nobilis*) have been reported to produce a calming effect by increasing behaviours indicative of relaxation (e.g., resting and sleeping) and also reducing barking in dogs housed in rescue shelters [32]. Pet Remedy (Unex Designs Ltd., Torquay, Devon, UK) is a commercial pharmacological intervention based on the herb valerian (*Valeriana officinalis*). Valerian has been shown to have anxiety reducing properties in humans [33,34], perhaps by enhancing production [35] or impeding breakdown [36] of the inhibitory neurotransmitter GABA (Gamma Amino Butyric Acid). This may occur because GABA receptors (at least in rodents) have an affinity with valerinic acid and valerinol [35,37,38].

We aimed to investigate whether Pet Remedy reduced behaviours indicative of stress in dogs already reported as suffering from stress-related disorders placed in a non-familiar “stressful” environment. We deployed a double-blind, placebo-controlled, and counterbalanced repeated measures design in order to account for individual variation in coping styles, sex, size and experience effects and any potential observer bias in scoring subjective behaviours. Given the range of possible indicators of stress, we considered a broad repertoire of behaviours, and compared overall levels of expression of these behaviours over the course of exposure to Pet Remedy or the placebo.

We predicted that Pet Remedy would reduce stress-affected behaviour in dogs placed in a non-familiar “stressful” environment and therefore, may have the capacity to improve canine welfare particularly for those suffering from stress-related disorders. We also expected to observe the strongest effects of Pet Remedy in dogs that suffer more profusely from stress-related behavioural problems (those individuals that express more hyper as opposed to hypo behaviours). Our predictions are based on previous research where Pet Remedies active ingredient valerian has previously been found to reduce stress in other species (e.g., Murphy et al., 2010).

## 2. Materials and Methods

### 2.1. Recruitment of Subjects

Owners of dogs were recruited by placing advertisements in local Veterinary Surgeries, pet shops, rescue shelters and the University of Exeter. The inclusion criteria were that dogs must be over 12 months of age, any breed or sex, not aggressive towards strangers and had previously shown signs of stress. Signs of stress-related behavioural problems were reported by the owner during the completion of an interview prior to the study taking place. All dogs participating in the study were described by their owners as showing anxious behaviour when in a new environment. Dogs were excluded if they were deemed so anxious or aggressive that they were unable to be handled safely. At the end of the trial owners received a free Pet Remedy trial kit as a result of their dogs participating in the study.

The study sample comprised of 28 dogs between the ages of 12 months and 11 years with a mean age of 4.64 years. Thirteen of these dogs were male (10 of which were neutered) and 15 female (12 of which were spayed). Five of the dogs were cross-breeds, other breeds included six King Charles Cavaliers, four Border Collies, three Lurchers, two Whippets, two Parsons Terriers and one of each of the following breeds; Standard Schnauzer, Jack Russell, Standard Poodle, Pomeranian, Yorkshire Terrier and Labrador Retriever. Dogs were from mixed backgrounds; including 16 from a rescue background. The experiment took place over a twelve week period between March and July 2013.

### 2.2. Experimental Environment

**Figure 1:**
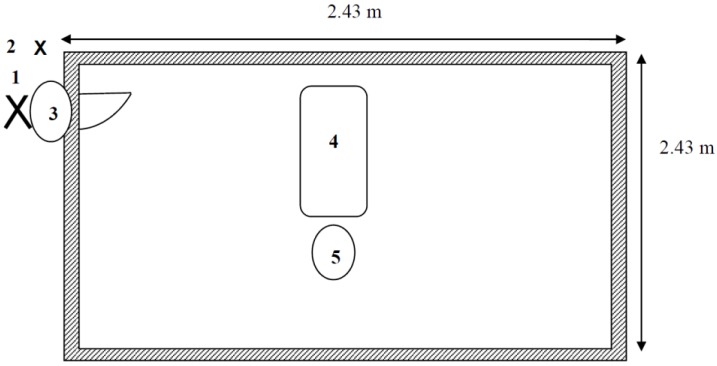
The experimental environment. **1** = Observer position, **2** = Site of the Pet Remedy/Placebo Plug-In Product, **3** = Video camera position, **4** = Plastic dog bed, **5** = Plastic water bowl.

The study took place in an enclosed room (3 m × 3 m) at the University of Exeter Streatham Campus, Psychology Department. The room contained a wooden holding pen (2.43 m × 2.43 m) with wipe clean flooring and walls, a plastic dog bed with washable blanket and a plastic water bowl and a diffuser to emit Pet Remedy or placebo into the room (Figure 1).

A novel room environment is considered to represent an acute stressor for dogs [28], particularly in the absence of conspecifics [39]. Dogs visited the experimental room on two occasions (approximately 7 days apart and between 10.00 and 17.30 h) and were exposed once to the placebo product and once to the Pet Remedy product. The placebo contained only the carrier agent for Pet Remedy, a volatising agent MMB (3-Methoxy-3-Methyl-1-Butanol). Pet Remedy consists of a concentration of 5.37% essential oils formulation (Valerian, Vetiver, Basil and Sage). The product can be administered via a spray, applied either on bedding, clothing or directly on to the animal’s coat or muzzle, or via an electronically heated plug-in diffuser which emits the product into the surrounding environment. We used the latter method of delivery. 

### 2.3. Experimental Procedure

Dogs were randomly allocated to an order of conditions. The diffuser was switched on 30 min prior to the focal dog entering the room to allow time for Pet Remedy or placebo to perfuse [32] with the conditions counterbalanced (placebo/Pet Remedy *n* = 15, Pet Remedy/placebo *n* = 13) to control for order effects. A two day “washout period” was instigated after each condition to allow the products to dissipate fully from the room and was aided by a built-in fan system. After each subject, blankets and bowls were changed and bed, walls and floor were wiped clean with a non-scented animal safe disinfectant spray to minimise individuals’ odours and spread of disease. The dog’s behaviour was filmed throughout the 30 min experimental period [40] using a video camera (Samsung, C20) which was hand-held by the observer and placed in a small hole cut in the holding pen. This allowed the camera to be positioned to enable full view of the focal dog. The observer was screened at all times in order to reduce effects on the dogs’ behaviour. Video data files were renamed by a naive assistant to ensure blind treatment of data during scoring. Footage was analysed at a later date with behaviours scored based on an ethogram (Table 1).

### 2.4. Behavioural Analysis

Fourteen behaviours were described as states and their duration measured (auto-grooming, digging, drinking, lying down, nosing, locomotion (walking and trotting), standing on hind legs, sitting, standing, circling, chewing, stretching, rearing and wall bouncing). A further nine behaviours were described as events and their number of occurrences were recorded (barking, howling, nose licking, paw lifting, sighing, whining, panting, yawning, urination/defecation). We recorded either the total durations (Table 2) or total occurrences of these behaviours (Table 3) during the 30 min of exposure to Pet Remedy or placebo.

A multivariate regression analysis (rank transformed) was conducted using the suite of 23 behaviours as a collection of dependent variables. Our independent variable was whether the dog was exposed to active Pet Remedy or a control substance from the diffuser. We also included “dog ID” and “order of conditions” as fixed variables. Further, we constructed a correlation matrix to check that none of the behaviours were highly correlated with each other. The results indicated that the behaviours were not highly correlated. We analysed the data with the statistics software IBM SPSS Statistics (version 20.0, SPSS Inc., Armonk, NY, USA, 2011).

### 2.5. Ethical Notes

All procedures were conducted according to ethical guidelines laid down by the University of Exeter Ethical Review Group (2013/202). Dogs were made as comfortable as possible by providing them with basic needs such as bedding and water. A cross-over within-subjects design was used and also a Russ Lenth power analysis [41] to determine how many dogs represented a population therefore reducing the number of dogs required. The study was sponsored by Unex Designs Ltd. (manufacturers of Pet Remedy, Torquay, Devon, UK) who paid for the tuition fees of Sienna Taylor and associated research costs. However, the experimental design, selection of subjects, data collection and analysis were conducted entirely independently of the Company or its representatives, with Joah Madden, who received no benefits from Unex Designs Ltd., ensuring impartiality throughout the research. 

## 3. Results

### Multivariate Regression Analysis

We conducted a multivariate regression analysis to investigate the influence of Pet Remedy compared to a placebo on the behaviour of dogs (for an overview see Table 4). In total we included 23 variables in the regression model. When considering the impact of Pet Remedy on the frequency of behaviours displayed by dogs, the model overall showed these variables were not statistically significant (F(9,41) = 0.805, *p* = 0.613, R^2^ = 0.14). The impact of Pet Remedy on the duration of behaviours displayed by dogs’ were also not statistically significant (F(14,41) = 0.669, *p* = 0.789, R^2^ = 0.18). We found no statistically significant influence of Pet Remedy on the behaviour variables (Table 4) although one variable, sighing, approached significance (Beta = −0.12, *p* = 0.052). 

## 4. Discussion

Stress relief products are widely used to manage behavioural problems in mammals. We investigated the efficacy of Pet Remedy, advertised as a natural stress relief product for mammals. In the current study, no statistically significant differences in behaviour were found in either the Pet Remedy or placebo condition. A lack of a discernible effect under either condition suggests that Pet Remedy did not affect behaviour of dogs’ placed in a potentially stressful non-familiar environment.

Although no statistically significant differences were found in the Pet Remedy or placebo condition, one behaviour, sighing, did approach significance (*p* = 0.052). Overall, dogs tended to sigh less in the Pet Remedy condition compared to the placebo condition. An increase in sighing behaviour has been proposed to indicate stress in dogs’ [39], thus suggesting that a reduction in sighing behaviour during the Pet Remedy condition may have indicated that dogs were less stressed. One interpretation is that Pet Remedy may have produced a calming effect allowing dogs to cope with the stressors that the non-familiar confined environment induced. Similar responses have been reported in previous studies [13,39] when dogs have been exposed to either social isolation or spatial restriction however, changes in sighing rate are usually recorded occurring alongside panting, lip licking and trembling [39]. It is important to note that there was a lack of support from a reduction in other indicators deemed as stress responses in dogs in the current study, and therefore this explanation should thus be viewed with caution. Moreover, an increase rather than decrease in deep respiration has also been proposed to indicate a relaxed state in dogs [42]. Sighing behaviour is also difficult to interpret with regard to stress, particularly in longer term studies [39]. For example, Beerda et al. (1999) only observed sighing in dogs during the first two weeks of spatially restricted housing and sighing was not observed for the full duration of the study. Therefore, sighing cannot be deemed as a reliable indicator of stress [39].

There are a number of limitations to this study. For example, age, size and breed could not be controlled for due to the high variance and relatively low sample size. These factors may have influenced the susceptibility of the dogs’ to Pet Remedy and the particular coping style of the dogs’. Variance in responses may also occur due to each individual’s prior experiences and particular stressors. Additionally, objective measures of physiological stress could have been collected to support the behavioural observations, including heart rate and levels of circulating cortisol. However, our pilot studies revealed that collecting salivary cortisol from stressed dogs was only sporadically successful, and that heart rates in unrestrained dogs were highly dependent on activity levels, making their interpretation problematic. A further limitation is the use of a within-subjects design which relies on the room being novel under both the placebo and Pet Remedy condition. However, to our knowledge, this is the first study to investigate the efficacy of Pet Remedy on the behaviour of dogs and therefore further research is required. We recommend that future studies use a larger sample size including a standardised breed type and weight to lower variance. Future studies could also incorporate a second novel room to overcome the issue of the same room not being deemed novel under both conditions. Moreover, a Likert scale accounting for how neophobic dogs are could also be incorporated so to avoid over or underestimating any potential effect of Pet Remedy.

The most effective, long term solution for behavioural problems is combining pharmacological treatments with behavioural modification programmes such as desensitisation training [25] therefore, to further this work future research should also focus on the effects of Pet Remedy used in combination with behavioural modification programmes.

## 5. Conclusions

In conclusion, our results suggest that Pet Remedy did not have a discernible effect on the behaviour of dogs placed in a non-familiar environment. This was the first study of its kind to investigate the efficacy of Pet Remedy and therefore, further research into the effects of Pet Remedy on the behaviour of dogs’ is required. Future research should concentrate on using a larger sample size more representative of a single breed and be investigated in combination with a behavioural modification programme.

## Figures and Tables

**Table 1:** Ethogram of behaviours, measured as frequencies (*f*) (events) or durations (*d*) (states).

Behaviour	Definition	Measurement Method *(f or d)*
Auto-grooming	Time spent in behaviours directed to own body including licking and scratching.	*d*
Digging	Time spent with one or both front paws in a digging motion, on floor or wall of holding pen.	*d*
Drinking	Time spent drinking water from bowl.	*d*
Lying down	Time spent ventral/lateral lying on ground with all four legs resting and in contact with ground. Eyes may be open or closed.	*d*
Nosing	Time spent investigating surroundings whilst sniffing and with nose held in contact with the edge of the holding pen surface.	*d*
Locomotion	Time spent in forward movement with legs resulting in shift of whole body to a new position in the pen.	*d*
Hind legs	Time spent with hind limbs in contact with the ground and fore limbs in air. Front legs do not touch pen sides or pen exit.	*d*
Sitting	Time spent with hind quarters on ground with two front legs being used for support.	*d*
Standing	Time spent with all four paws on ground and legs upright and extended supporting body.	*d*
Circling	Time spent in circling locomotion, often repetitive (performed more than once).	*d*
Chewing	Time spent chewing non-nutritive material.	*d*
Stretching	Time spent with front or hind legs outstretched.	*d*
Exit “rear”	Time spent standing on hind legs with front legs resting or digging against exit.	*d*
Wall bouncing	Time spent standing on rear legs with front legs rebounding off, resting on or digging on pen walls other than exit. Often repetitive (performed more than once).	*d*
Barking	Number of times dog makes staccato vocalisation.	*f*
Howling	Number of times dog makes loud prolonged vocalisation with mouth open and head extended.	*f*
Nose licking	Number of times dog extends its tongue upwards to cover nose, before retracting into mouth.	*f*
Paw lifting	Number of times dog raises a single paw and holds it above the ground without moving.	*f*
Sighing	Number of times dog intakes a deep breath and blows out loudly.	*f*
Whining	Number of times dog makes high pitched vocalisation.	*f*
Panting	Frequency of episodes spent with mouth open with tongue extended accompanied with rapid breathing and expansion/contraction of chest.	*f*
Yawning	Number of times dog opens its mouth wide open for a period of a few seconds and then closes.	*f*
Urination/defecation	Number of times dog either urinates or defecates in the pen.	*f*

**Table 2:** The mean (±S.D.) duration (seconds) of each behaviour displayed by the dogs in the Pet Remedy and placebo conditions.

Behaviour	Pet Remedy	Placebo
Auto-grooming	5.00 (16.553)	2.50 (7.188)
Digging	7.25 (18.336)	7.75 (18.440)
Drinking	7.86 (11.629)	3.32 (7.329)
Lying down	588.21 (527.466)	761.86 (632.069)
Nosing	13.64 (19.383)	18.82 (31.852)
Locomotion	187.07 (172.500)	120.75 (131.698)
Hind legs	1.04 (2.168)	0.82 (1.926)
Sitting	264.79 (268.978)	345.64 (429.699)
Standing	676.29 (472.696)	502.71 (500.408)
Circling	6.96 (36.071)	3.86 (19.084)
Chewing	1.93 (5.799)	2.32 (9.888)
Stretching	2.61 (10.843)	2.18 (11.528)
Exit Rear	31.43 (77.087)	21.96 (52.814)
Wall Bounce	5.93 (12.936)	5.50 (15.820)

**Table 3:** The mean (±S.D.) frequency each behaviour was displayed by the dogs in the Pet Remedy and placebo conditions.

Behaviour	Pet Remedy	Placebo
Barking	121.75 (265.760)	105.00 (242.474)
Howling	2.11 (10.038)	1.82 (9.638)
Nose Licking	24.14 (26.661)	52.54 (126.948)
Paw Lifting	0.93 (2.581)	1.14 (2.851)
Sighing	0.71 (1.084)	1.21 (1.424)
Whining	86.32 (126.251)	121.93 (151.436)
Yawning	2.14 (3.015)	3.11 (5.101)
Urination/defecation	0.21 (0.957))	0.04 (0.189)
Panting	07.89 (13.048)	8.57 (15.683)

**Table 4:** Results of the multivariate regression analysis.

Behaviour	Beta	Std Error	Sig.
Barking	−0.00	0.01	0.701
Howling	0.23	0.18	0.213
Nose Licking	−0.00	0.00	0.525
Pawing	−0.05	0.05	0.397
Sighing	−0.12	0.06	0.052
Whining	−0.00	0.00	0.183
Yawning	−0.00	0.02	0.836
Urination/Defecation	0.13	0.23	0.573
Panting	0.00	0.01	0.648
Auto-grooming	0.01	0.04	0.640
Chewing	−0.00	0.05	0.900
Circling	0.02	0.11	0.842
Digging	0.01	0.02	0.534
Drinking	0.02	0.02	0.192
Exit Rear	0.00	0.01	0.544
Hind Legs	−0.09	0.09	0.312
Locomotion	0.00	0.00	0.245
Lying Down	0.01	0.01	0.296
Nosing	−0.01	0.01	0.161
Sitting	0.00	0.00	0.358
Standing	0.01	0.01	0.186
Stretching	0.05	0.11	0.655
Wall Bounce	−0.01	0.03	0.680
